# Predictors of oral health-related quality of life in 2–5 year-old children in the South of Iran

**DOI:** 10.1186/s12955-020-01587-7

**Published:** 2020-12-11

**Authors:** Masoumeh Abbasi-Shavazi, Elham Mansoorian, Sara Jambarsang, Amene Hosseini-Yekani, Vahid Rahmanian

**Affiliations:** 1 Department of Health Education and Promotion, School of Public Health, Shahid Sadoughi University of Medical Sciences, Yazd, Iran; 2 Department of Health Education and Promotion, School of Public Health, Shahid Sadoughi University of Medical Sciences, Yazd, Iran; 3 Research Center of Prevention and Epidemiology of Non-Communicable Disease, Department of Biostatistics and Epidemiology, School of Public Health, Shahid Sadoughi University of Medical Sciences, Yazd, Iran; 4 School of Dentistry, Shahid Sadoughi University of Medical Sciences, Yazd, Iran; 5 Research Center for Social Determinants of Health, Jahrom University of Medical Sciences, Jahrom, Iran

**Keywords:** Quality of life, Preschool child, Educational models, Structural equation modelling, Jahrom

## Abstract

**Background:**

Dental and oral diseases can have negative impacts on children’s quality of life. The aim of this study was to determine the predictors of oral health-related quality of life (OHRQoL) in the children aged 2–5 years old.

**Materials and methods:**

A total number of 288 children aged between 2 and 5 years were selected and stratified by gender from three community health centres located in the city of Jahrom, south of Fars Province, Iran. The data collection tool was a researcher-made questionnaire whose validity and reliability was confirmed. The questionnaire was completed by parents/caregivers of the children. A multiple linear regression analysis was performed with quality of life as the dependent variable and, based on covariance structural analysis, evaluated the goodness of fit of the resulting structural equations models.

**Results:**

The results showed that predisposing factors with a coefficient of 0.0457 (*p* = 0.015) and reinforcing factors ones with a coefficient of 0.2748 (*p* < 0.001) were correlated with the oral health behaviours. Moreover, there was a relationship between such behaviours with a coefficient of 0.1612 (*p* < 0.001) and oral health status and the given status with a coefficient − 0.9714 was correlated with OHRQoL (*p* < 0.001). Based on the covariance structural analysis, the resulting model was found to exhibit a reasonable goodness of fit.

**Conclusion:**

The predictors of the children’s OHRQoL included predisposing, strengthening, oral health behaviours and oral health status. Therefore, planning to enhance supportive family behaviours and to boost predisposing factors including knowledge, attitudes, perceived benefits, and self-efficacy in parents and their oral health behaviours is recommended.

## Introduction

Dental and oral diseases can have negative impacts on children’s health and mental well-being as well as their families. It can also cause pain and discomfort in this age group. Furthermore, the given diseases can put limits on the activities of individuals at school, or home and consequently waste significant time of study throughout the world [1, 2] .

Dental caries is the most commonly reported oral/chronic health disease in children [3, 4]. This disease has been further recognized as a pandemic whose prevalence rates in children in the European countries, the UK, Japan, Asia, Africa, the Middle East, and Indonesia, have been reported by 40%, 12%, 25%, 36–85%, 38–45%, 22–61%, and 90% respectively. The given values in Iran, Senegal, and Thailand have been also at the range of 50–60% [5].

The World Health Organization (WHO) recognizes dental health and oral hygiene as a one which is a part of general health during the lifetime. They also acknowledged that poor dental health and oral hygiene, as well as untreated illnesses, can have their own profound impacts on quality of life (QoL) in the communities [6].

QoL is a broad concept that is complexly affected by physical, mental and social health [7]. Evaluating the impacts of dental health and oral hygiene on QoL, especially in young children, is of utmost importance because dental and oral conditions can affect growth, weight, self-confidence, socialization, learning abilities and daily activities of children and parents [8, 9]. Moreover, treatment of oral disease is extremely costly and is ranked as the fourth most expensive disease to treat in most developed countries. In low-income countries, if treatment was available, the costs of dental caries alone in children would exceed the total health care budget for children.[10].

The HRQoL was introduced as a multidimensional subject containing physical, social, and psychological dimensions. The given concept indicates if people are satisfied with their dental health status and if their functioning has been affected or disrupted by this condition [11]. OHRQoL is vital in children because dental and oral diseases are so common in this age group and problems caused by these diseases, especially dental pains or visual problems can have negative impacts on QoL in children at present and the future. It also impacts on their daily routine activities including playing, sleeping habits, nutrition, social participation, and academic performance [12]. In this regard, the findings of the study by Jabbarifar et al. [13] pointed out the unacceptable status of OHRQoL in Iranian children.

As the ultimate goal in children’s dental health and oral hygiene is to improve their QoL, the use of a model as a framework for identifying factors related to OHRQoL is important. In this respect, PRECEDE (predisposing, reinforcing, and enabling factors) model provides a systematic process for health planning and evaluation [14]. The predisposing factors are awareness, attitudes, beliefs, convictions, and perceptions of an individual or population that facilitate or hinder motivation to change. The reinforcing factors are related to the reaction of others to a person’s behaviour, which can be rewarding or punishing. The enabling factors can manifest themselves as barriers and skills [15]. The capacity of this model to plan, expand, and evaluate interventions has been verified in other studies [16–18].

PRECEDE phase constitutes of five stages: (1) social diagnosis, (2) epidemiological diagnosis, (3) behavioural and environmental diagnosis, (4) educational and organizational diagnosis and (5) administrative and political diagnosis [18]. Applying the actions outlined in stages 1–5 of the PRECEDE phase to perform a cross-sectional survey in a combination of a literature review tried to determine the predictors of OHRQoL in preschool children.

It should be noted that mothers are considered as the main caregivers performing dental health and oral hygiene measures in children aged 1 to 3 years old. Even though the development of children in the domain of oral health and dental hygiene starts in pre-school period (aged 3 to 6 years), mothers are still the main caregivers meeting dental health and oral hygiene needs in children [19]. Therefore, it seems that it is necessary to reflect on predisposing, enabling, and reinforcing factors associated with mothers in terms of evaluating QoL in children [20]. To our best knowledge, no specific study has examined the factors related to OHRQoL in children aged 2–5 years based on this model. Therefore, the purpose of the present study was to determine the predictors of oral health-related quality of life (OHRQoL) in the children aged 2–5 years old.

## Materials and methods

### Study design and participants

This cross-sectional study was conducted on a total number of 288 children aged 2 to 5 years. The children referred, by their parents, to the community health centres in the city of Jahrom located in the county of Jahrom, Fars province, Iran, to receive Primary Health Care (PHC) services. The PHC services in response to the global strategy for health for all by the year 2000 is defined. The PHC services in the health system was introduced in Iran in 1985. The PHC services in this system are free of charge.

The sample unit was selected and stratified by gender from three community health centres. The required sample sizes were calculated using a similar study [16] including 95% confidence level, 6.8 standard deviation and 0.78 margin of error.

The inclusion criteria in this study were an agreement to participate in the study as well as the presence of baby teeth in a child and the absence of permanent ones in children. These children were required to be geographically located inside the area under the coverage of healthcare services provided by community healthcare centres. This study has excluded parents (fathers, mothers, or child caregiver) who were not willing to participate in the study, children and parents who disliked clinical examinations. The exclusion was also extended to the children aged 2–5 years who had previously received dental and oral healthcare services (varnishing and fluoride therapy) in private kindergartens and private dental clinics.

The data collection instrument was a questionnaire comprised of three parts; demographic characteristics (child’s age, gender, birth order, number of children in a family, parents’ age, education and occupation, child-parent relationships, and the household income), OHRQoL scale, and constructs of PRECEDE model associated with OHRQoL in children aged 2 to 5 years. To determine the validity of the questionnaire, Content Validity Ratio (CVR) and Content Validity Index (CVI) were used and ultimately items with CVR of higher than 0.99 and CVI of over 0.78 were chosen. Furthermore; adherence to grammar, use of proper words, the importance of items, positioning items in suitable places, and necessary time to complete the questionnaire were evaluated and approved by experts of health education. Moreover, the internal validity or reliability of the designed instruments was confirmed by calculating the Cronbach’s alpha coefficient over 0.72, about the cut-off point of 0.65 [21] (Additional file 1).

### Data collection instrument

To evaluate OHRQoL, we used the Early Childhood Oral Health Impact Scale (ECOHI). This instrument has translated and culturally adapted to assess OHRQoL of preschool children in some communities [22–25]. Jabarifar et al. [22] translated this questionnaire into Persian and Proved in a study in 2–5 years old children, that the Persian version of ECOHI for assessing OHRQoL of preschool children has appropriate validity and reliability.

The research instrument is comprised of 13 items in two general categories; the first 9 items address children’s oral health status and its impacts including symptoms of the disease, child’s functioning, mental-psychological health status, self-confidence, and social interactions. The last four items examine the effect of children’s oral health status on their families, which consists of family concerns and its functions. Each answer to these 13 items was scored based on Likert scale, score one (never) to score five (frequently). The higher scores attributed to each child indicated lower OHRQoL. Moreover, answers to items with “I don’t know” as well as unanswered items were considered as mean total scores obtained by each individual from one to 13. Generally, the total scores of this index have range of 13–65, which the scores range 13–29 good, 30–47 fair and 48–65 “poor” [22].

In the next step, the questionnaire was designed based on the PRECEDE model. The PRECEDE variables included oral health status, oral health behaviours, predisposing factors, reinforcing factors and enabling factors, which are described as follows:

Self-report questionnaire (researcher-made) for oral health status was also utilized to evaluate the oral health status. The questionnaire consists of four questions including assessing the child’s oral health status from the parents’ point of view (very bad/bad/good/very good/I do not know), awareness of decayed teeth (yes/no/not sure), frequency of dental caries and cleaning teeth after brushing (yes/no/I don’t know) which was ultimately scored between 0 and 7. The lower score obtained from this questionnaire indicated the lower oral health status.

To evaluate oral health behaviours, a questionnaire with seven items was developed in which children’s brushing behaviours, parental monitoring methods used for children’s brushing behaviours, sugar consumption (chocolate, sweets, and sweetened beverages) by children and limiting these substances by parents included. The visits to dentists and gaining information about dental and oral health were also evaluated with scores ranged between 0 and 16. In this respect, a higher score could reflect more favourable oral health behaviours in the children.

To assess the predisposing factors, the variables of knowledge, attitude, perceived barriers, perceived benefits, parental self-efficacy, oral health behaviour of parents were evaluated.

Parental knowledge about children’s dental health status was evaluated through a seven item questionnaire scored from zero to seven in which a higher score indicated more knowledge. Parents’ attitudes towards children’s dental health status were also evaluated by 18 items with a three-points Likert-type scale including “disagree”, “neutral”, and “agree” and scored from 18 to 54 in which a higher score showed more favourable attitudes. Perceived barriers to children’s dental health and oral hygiene were evaluated using 16 items with a three-points Likert-type scale including “at all”, “somewhat”, and “often”. The scores ranged from zero to 32 and a higher score reflected more perceived barriers by parents. Nine items with a three-points Likert-type scale from “disagree”, “neutral” and “agree” were utilized to evaluate perceived benefits associated with dental health and oral hygiene. The scores ranged between 9 and 27 and a higher score represented higher perceived benefits by the parents. Parental self-efficacy regarding dental health and oral hygiene was also evaluated by six items using a 3-point Likert-type scale of “at all”, “somewhat”, and “often” scored between zero to 12 in which higher scores showed more parental self-efficacy. To evaluate the oral health behaviours in parents; a four-item questionnaire was used which ranged between zero to 12 and the higher scores indicating better functioning and welfare.

The enabling factors were similarly evaluated using 11 items with a 3-point Likert-type scale from “never”, “somewhat”, “often” with a score range from zero to 22 in which higher scores indicated more access to dental health and oral hygiene resources and amenities. Furthermore, the reinforcing factors were evaluated by assessing supportive family behaviours via six items with a three-point Likert-type scale ranging from “at all” to “often”, scored between zero to 12, in which higher scores showed more support by spouse and children.

### Statistical analysis

After collecting the data and coding the questionnaires, they were imported into the Stata software (version 14) and then analysed using descriptive (frequency tables, mean and standard deviation) and analytical (Kolmogrov–Smirnov test for check the normality, Kruskal–Wallis tests) statistics. Multiple linear regression analysis was necessary to elucidate the factors by which the dependent variables might potentially be influenced, and to what extent. In the next step, structural equation modelling (SEM) was employed to examine the relationships between the PRECEDE model constructs.

In this model, three steps were considered as follows: In step 1, Oral health behaviours were considered as a dependent variable and predisposing, reinforcing and enabling factors were considered as an independent variable. Under step 2, Oral health status was considered a dependent variable and oral health behaviours was considered an independent variable and in the third step OHRQoL was considered as a dependent variable and oral health status was considered as an independent variable.

It should be noted that SEM is a general and strong multivariate analysis technique from the regression family, i.e., it is an extension of general linear model testing a set of regression equations simultaneously. The goodness of fit of the model was evaluated based on the χ^2^, goodness of fit index (GFI), adjusted goodness of fit index (AGFI). Acceptable goodness of fit was defined as χ^2^/*df* < 3.0, GFI > 0.90, AGFI > 0.85 [23]. The significance level was also considered by 0.05.

## Results

The total number of 288 children referred to community health centres to receive PHC services were enrolled in this study. As summarised in Table 1, 238 (82.6%) of the questionnaires were completed by the mothers. In terms of the age group the classification was as follows: 73 children (25.3%) between 24 and 25 months old, 81 of them (28.1%) aged 36–47 months old, 79 of them (27.4%) aged between 48 and 49 months old, and 55 of them (19.2%) aged between 71 and 60 months. Concerning the gender, 148 girls (51.4%) and 179 children (62.2%) were the first-born babies in their family. In terms of the number of children in a family, 112 children (38.9%) were only-child and 87 of them (30.2%) were the second-born. Considering parental age, the highest frequency of father’s age was 208 individuals (72.2%) in the age group of 31–40 years and for the case of mother’s age that was 159 individuals (55.2%) for the age group of 31–40 years. The highest level of education among fathers included 105 individuals (36.5%) with high school diploma and the given frequency was 109 mothers (37.8%) holding bachelor’s degrees (see Table 1).

**Table 1:** Frequency distribution of demographic variables of the study participants

Variable	Item	Absolute frequency	Relative frequency (%)
Respondents	Mother	238	82.6
	Father	46	16
	Child’s caregiver	4	1.4
Child’s age	24–35 months	73	25.3
	36–47 months	81	28.1
	48–59 months	79	27.4
	60–71 months	55	19.2
Child’s gender	Female	148	51.4
	Male	140	48.6
Birth order	First-born	179	62.2
	Second-born	87	30.2
	Third-born and higher	22	7.6
Number of children in a family	Only child	112	38.9
	2–3 children	160	55.6
	4–5 children	16	5.5
Parental age			
Father	Below 30 years old	30	10.4
	31–40 years old	208	72.2
	41–50 years old	44	15.3
	Over 50 years old	6	2.1
Mother	Below 30 years old	121	42
	31–40 years old	159	55.2
	41–50 years old	8	2.8
Level of education			
Father	Primary school	18	6.2
	Middle school	53	18.4
	High school diploma	105	36.5
	Associate’s degree	21	7.3
	Bachelor’s degree	67	23.3
	Master’s degree and higher	24	8.3
Mother	Primary school	12	4.2
	Middle school	19	6.6
	High school diploma	106	36.8
	Associate’s degree	23	8
	Bachelor’s degree	109	37.8
	Master’s degree and higher	19	6.6
Father’s occupation	Employee	82	28.5
	Worker	37	12.8
	Self-employed	159	55.2
	Retired	2	0.7
	Other	8	2.8
Mother’s occupation	Housewife	237	82.3
	Work from home	4	1.4
	Employed outside the home	42	14.6
	Other	5	1.7

Moreover, a significant difference was observed between the mean score of oral health behaviours in terms of age groups (*p* = 0.001), but such a difference was not significant based on gender (*p* = 0.60). Besides, there was no difference in the mean scores of OHRQoL, oral health status, predisposing factors, reinforcing factors, and enabling factors in terms of age and gender groups groups (*p* > 0.05) (see Table 2).

**Table 2:** Mean and standard deviation of OHRQoL, oral health status, and PRECEDE model constructs in terms of age and gender groups of children in the present study

Mean score	Age groups (months)										*p* value	Total mean score
	24–35		36–47		48–59		60–71		Total			
	Girl	Boy	Girl	Boy	Girl	Boy	Girl	Boy	Girl	Boy		
OHRQoL	18.45 ± 7.71	18.13 ± 9.34	18.80 ± 8.39	17.50 ± 5.69	20.73 ± 9.51	20.98 ± 9.26	21.16 ± 9.22	20.64 ± 6.92	19.69 ± 8.70	19.21 ± 8.04	0.89	19.46 ± 8.38
Oral health status	4 ± 1.79	3.78 ± 2.29	3.76 ± 2.12	3.62 ± 180	3.55 ± 2.21	3.35 ± 1.97	3.41 ± 2.20	3.75 ± 2.01	3.70 ± 2.07	3.61 ± 2	0.76	3.66 ± 2.04
Oral health behaviors	6.86 ± 2.66	6.58 ± 3.03	6.88 ± 1.90	8.15 ± 2.66	8.29 ± 2.35	7.65 ± 2.50	8.59 ± 2.25	8.57 ± 2.54	7.59 ± 2.40	7.70 ± 2.77	0.001	7.64 ± 2.58
Predisposing factors	96.51 ± 8.91	97.27 ± 7.50	96.07 ± 6.70	97.41 ± 7.58	96.69 ± 6.90	95.78 ± 9.63	96.07 ± 6.12	98.32 ± 6.60	96.35 ± 7.21	97.12 ± 7.94	0.97	96.73 ± 7.57
Reinforcing factors	7.35 ± 2.32	7.61 ± 2.23	8 ± 2.72	7.51 ± 2.44	8.29 ± 2.42	7.92 ± 2.91	8.44 ± 2.19	8.54 ± 1.93	8 ± 2.45	7.85 ± 2.44	0.08	7.93 ± 2.44
Enabling factors	11.08 ± 3.59	11.42 ± 4.02	11.67 ± 4.44	10.82 ± 3.64	12.33 ± 3.55	12 ± 4.46	12.44 ± 4.06	11.86 ± 3.71	11.85 ± 3.92	11.49 ± 3.97	0.24	11.68 ± 3.94

**Fig. 1:**
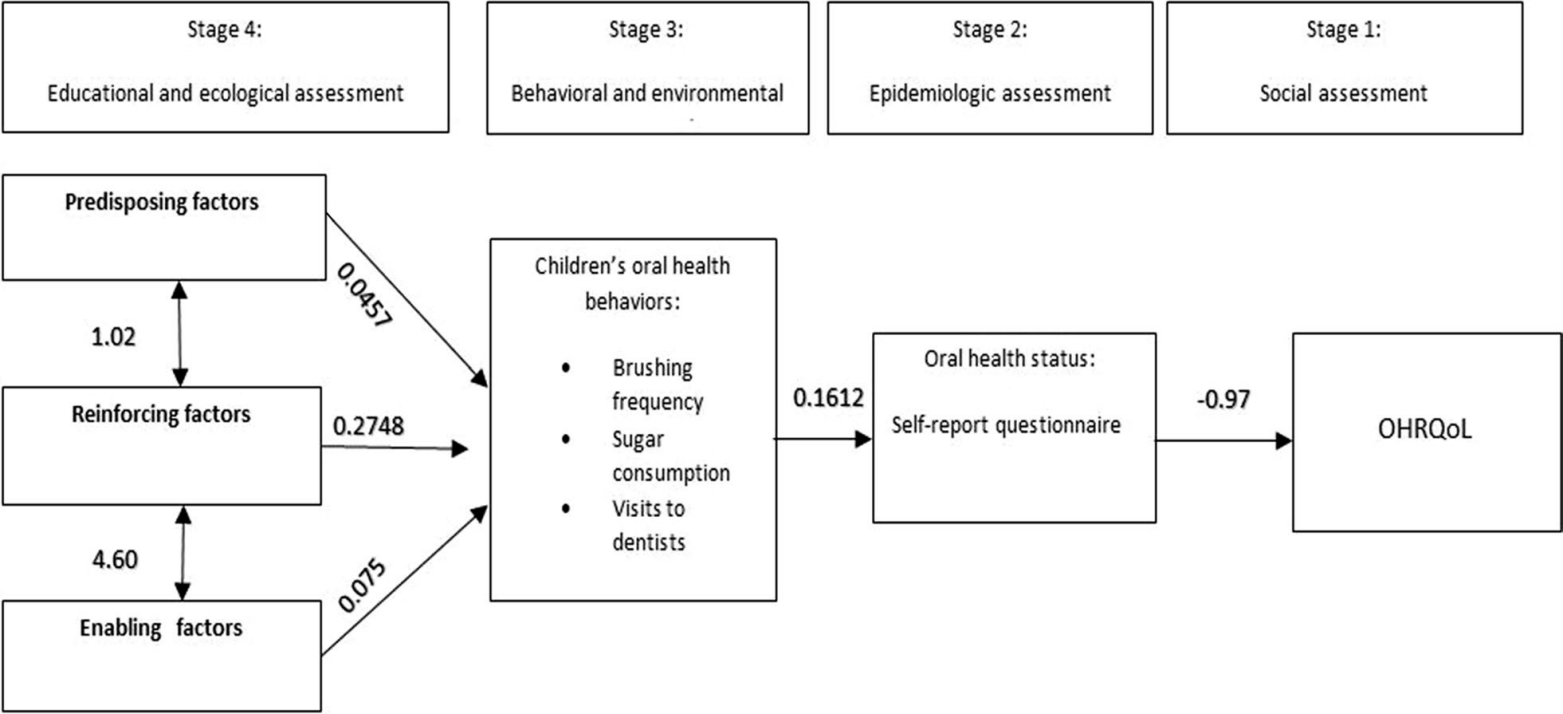
Predictors of OHRQoL in children based on the results of path analysis of PRECEDE model constructs

The PRECEDE model proportional to the study population resulted from the SEM-based path analysis is illustrated in Fig. 1.

The findings of this model showed that the predisposing factors with a coefficient of 0.0457 were correlated with oral health behaviours (*p* = 0.015). There was also the correlation between reinforcing factors with a coefficient of 0.2748 and oral health behaviours (*p* < 0.001). The oral health behaviours with a coefficient of 0.1612 were also correlated with oral health status (*p* < 0.001). Moreover, a correlation was observed between oral health status with a coefficient of 0.9714 and OHRQoL (*p* < 0.001) (see Table 3). Measures of model fit (χ^2^/*df* = 1.475, GFI = 0.916, AGFI = 0.863, indicated that the model had an acceptable goodness of fit.

**Table 3:** SEM results for examining relationships between PRECEDE model constructs

	SEM coefficient	Standard deviation	Significance level	95% confidence interval	
				Low	High
Oral health behaviours					
Predisposing factors	0.0457	0.0188	0.015	0.0088	0.0826
Reinforcing factors	0.2748	0.0659	0.001	0.1455	0.4041
Enabling factors	0.0750	0.0410	0.068	− 0.0054	0.1555
Oral health status					
Oral health behaviours	0.1612	0.0445	0.001	0.07196	0.2504
OHRQoL					
Oral health status	− 0.9714	0.2349	0.001	− 1.431	− 0.5109
Covariance for predisposing and reinforcing factors	1.025	1.090	0.347	− 1.111	3.162
Covariance for predisposing and enabling factors	2.917	1.763	0.098	− 0.539	6.373
Covariance for reinforcing and enabling factors	4.601	0.628	0.001	3.369	5.833

## Discussion

The purpose of this study was to determine the predictors of OHRQoL in children aged 2–5 years using the PRECEDE model. Based on the findings of this study, the predictors of the children’s OHRQoL included predisposing, strengthening, oral health behaviours and oral health status.

Based on the findings of this study, the mean score of OHRQoL in children was reported good which was consistent with the results of the study by Jabbarifar et al. [23] in the city of Shiraz as the capital of Fars Province, Iran. This consistency may be attributed to the closeness of Shiraz and Jahrom where the people almost follow the same pattern in terms of culture and diet style. Moreover, children’s oral health status in this study was evaluated at a moderate level which was in line with the findings of the investigation by Ghazanfari et al. [20] on 3- to 6-year-old children in the city of Ilam, Iran. This implies that the parents had evaluated their children’s status in terms of dental caries and satisfaction with cleaning as well as their dental health and oral hygiene at a moderate level (within due or reasonable limits).

In the present study, the mean score of oral health behaviours in children was lower than the average. In this respect, the findings of this study were consistent with those reported by Nokhostin et al. [24], Dehvari et al. [25] and Yavari et al. [26]. As the evaluation of oral health behaviours in this study included brushing, sugar consumption, and visits to dentists and because children in this age group follow mainly their parents, therefore empowering parents through providing information and education in the domain of dental health oral hygiene and teaching skills can help to improve oral health behaviours for the children.

Besides, the results of this study showed that the oral health behaviours in children were significantly correlated with predisposing factors which were in line with the findings of Malek Mohammadi et al. [27] focused on the city of Kerman which is the neighbouring province to Fars. Moreover, these results confirmed the PRECEDE model assumption implying a direct relationship between predisposing factors and behaviours. Since predisposing factors in this study were evaluated through knowledge, attitudes, perceived barriers, benefits, parental self-efficacy, and the oral health behaviours by parents, the relationship between predisposing factors and behaviours indicated that promotion of each factor could lead to improvement of the oral health behaviours in the children in three domains of brushing, sugar consumption, and visits to dentists. In this line, the study by Sahraie et al. [28] also showed that maternal knowledge and beliefs about brushing could be among factors acting as barriers to brushing behaviours in children. Wong et al. [29] studied of OHRQoL in Hong Kong focused on preschool children. They showed that parents of young children with dental caries experience, both the children and other family members had poorer quality of life.

The results of this work show that there is a significant relationship between the oral health behaviours in children and reinforcing factors which was confirmed by the PRECEDE model. The reinforcing factors in this model included supportive behaviours by spouse and other children which were in line with the findings of Sahraie et al. [28]. They emphasized the role of family members in children’s dental health and oral hygiene and stressed that it is the most important perceived barrier for mothers of 3- to 6-year old children.

It should be noted that the oral health behaviours were not significantly correlated with enabling factors which were in contrast with the findings of the study by Binkley et al. [30] in which dental health and oral hygiene in individuals was significantly correlated with the mental disabilities. There was also a significant relationship between growth impairment and enabling factors which were consistent with the assumption raised by PRECEDE model. In the present study, assessing the enabling factors such as the skill needed to brush a child’s teeth, preparing toothbrushes and toothpaste, the cost of transportation for child care and oral care, the cost of dental examinations, insurance payments, health centre staff training done to parents. Measuring enabling factors in a more limited and specific way in the field of children's oral health can be more appropriate.

Other results of this study showed that oral health behaviours were significantly correlated with oral health status. In this regard, the findings of the study by Moalemi et al. [31] conducted in the city of Tehran, Iran, suggested that the oral health behaviours in 9-year-old primary school children were correlated with the oral health status. Our results were in agreement with the PRECEDE model assumption in which a direct relationship between predisposing factors and behaviours had been highlighted. Since the oral health status, frequency of dental caries, and dental health and oral hygiene in children were evaluated from the perspectives of parents in this study, the presence of such a relationship could imply that the way parents had evaluated children’s brushing, sugar consumption, and visits to dentists could influence their oral health status.

Based on the findings of this work, the oral health status had a direct impact on OHRQoL. It means that improving the oral health status could enhance OHRQoL which was consistent with the findings of the study by Saleki et al. [32] in Iran. Saho et al. [33] in the study of structural equation modelling to detect predictors of oral health-related quality of life in Japan showed that oral health status was directly associated with the OHRQoL. Furthermore, Rodolfo et al. [1] reported that dental caries, mobile milk teeth, tooth position, bleeding gums, and bad breath were associated with the wors OHRQoL children in Rio de Janeiro. Moreover, the given results confirmed the PRECEDE model assumption underlining a direct relationship between predisposing factors and the behaviours.

Based on the results of this study, reinforcing and enabling factors were significantly interrelated, confirming the PRECEDE model assumption in which interrelationship between reinforcing and enabling factors was confirmed. Therefore, planning interventions for strengthening any of the above factors could lead to mutual improvement. Additionally, educating parents as well as providing them with the right information about the areas of oral/dental health and also educate them the required skills as predisposing factors could lead to improve the reinforcing factors.

Binkley et al. [30] in the study of using PRECEDE–PROCEED planning model in designing an oral health strategy united states, showed that PRECEDE–PROCEED planning model can be used for planning, development and evaluation of interventions in oral health.

The strengths of the present study are its exploratory approach towards designing the research instrument in which CVR and CVI were measured as a tool to evaluate parents’ perceptions more objectively.

The limitations of this study were the self-assessing of life-scale related to the oral health and other factors related to the pattern asked by the parents which can be effective in external validity the results of this study. Therefore, evaluating dental health status using objective scales including dmft and Modified Gingival Index (MGI) could have positive impact on making these studies and their results more objective. Also, the outcome of this study can be generalized that the quality of life associated with oral health for the children who contact the health centres for primary health care services may be different from those who do not receive these care services.

## Conclusion

The predictors of the children's OHRQoL included predisposing, strengthening, oral health care behaviours and oral health status. Therefore, proper planning to improve family behaviours and strengthening the predisposing factors such as knowledge, attitudes, perceived benefits, parental self-efficacy and their oral health status, as well as reducing perceived barriers can enhance children’s oral health behaviours and consequently promote the oral health status and OHRQoL in children aged 2–5 years. Considering elements of the PROCEDE model in interventions aimed at enhancing oral health status in children might be valuable and therefore, worth investigating in future research.

## Supplementary information

**Additional file 1**. Early Childhood Oral Health Impact Scale (ECOHI).

## Data Availability

The data are available upon request from the corresponding author.

## References

[1] Castro RA, Portela MC, Leão AT, de Vasconcellos MT (2011). Oral health–related quality of life of 11-and 12-year-old public school children in Rio de Janeiro. Commun Dent Oral Epidemiol.

[2] Mohammadi M, Vaisi A, Jalali R, Ghobadi A, Salari N (2018). The prevalence of dental caries in deciduous and permanent teeth in Iranian children: a systematic review and meta-analysis. J Res Dent Sci..

[3] Mouradian WE, Slayton RL, Maas WR, Kleinman DV, Slavkin H, DePaola D, Evans C, Wilentz J (2009). Progress in children's oral health since the surgeon general's report on oral health. Acad Pediatr.

[4] Hazavehei SMM, Shirahmadi S, Taheri M, Noghan N, Rezaei N (2015). Promoting oral health in 6–12 year-old students: a systematic review. J Educ Commun Health.

[5] WHO. Oral health. https://www.who.int/oral_health/publications/factsheet/en/. Accessed 20 Feb 2019.

[6] Torabi M, Karimi A, Sheikhzadeh A, Karimi AM (2009). Assessment of oral health indices in Kerman adults aged 35–44 years. J Isfahan Dent Sch.

[7] Akhter R, Hassan NMM, Martin EF, Muhit M, Smithers-Sheedy H, Badawi N, Khandaker G (2019). Caries experience and oral health-related quality of life (OHRQoL) of children and adolescents with cerebral palsy in a low-resource setting. BMC Oral Health.

[8] Tomazoni F, Vettore MV, Mendes FM, Ardenghi TM (2019). The Association between sense of coherence and dental caries in low social status school children. Caries Res.

[9] Amirabadi F, Saravani S, Miri-Aliabad G, Khorashadi-Zadeh M (2019). The Association between dental health status and oral health-related quality of life of children diagnosed with β-Thalassemia Major in Zahedan City, Iran. Int J Pediatr.

[10] Suprabha BS, Rao A, Shenoy R, Khanal S (2013). Utility of knowledge, attitude, and practice survey, and prevalence of dental caries among 11- to 13-year-old children in an urban community in India. Glob Health Action.

[11] Hillebrecht A-L, Hrasky V, Anten C, Wiegand A. Changes in the oral health-related quality of life in adult patients with intellectual disabilities after dental treatment under general anesthesia. Clin Oral Investig 2019:1–9. 10.1007/s00784-019-02820-4 30707300

[12] Masumo RM, Ndekero TS, Carneiro LC (2020). Prevalence of dental caries in deciduous teeth and oral health related quality of life among preschool children aged 4–6 years in Kisarawe. Tanzan BMC Oral Health.

[13] Jabarifar SE, Khadem P, Ahmadi S, Hajiahmadi M, Nilchian F (2011). Assessment of psychometric projection of the Persian version of the Child Perception Questionnaire (CPQ8-10) in 8–10 year-old students in Isfahan. J Isfahan Dent Sch.

[14] Mohammadizeydi E, Farmanbar R, Asadpur M, Noroozi A, Rahmati F, Ghazanfari Z, Aamidi M, Rahnama P, Ghofranipur F (2006). The precede–proceed model. Pattern change behavior.

[15] Green L, Kreuter M (2005). Health program planning: an educational and ecological approach.

[16] Golkari A, Moeini A, Jabarifar SE (2014). Relationship of socioeconomic status with quality of life related to oral and dental health of 2–5-year-olds in Shiraz. J Isfahan Dent Sch.

[17] Nadrian H, Morowatisharifabad MA, Bahmanpour K (2011). Development of a rheumatoid arthritis education program using the PRECEDE_PROCEED model. Health Promot Perspect.

[18] Nadrian H, Morowati Sharifabad MA, Soleimani SH (2010). Paradims of rheumatoid arthritis patients quality of life predictors based on path analysis of the Precede model. Hormozgan Med J.

[19] Naderifar M, Akbarsharifi T, Pairovi H, Haghani H (2006). Mothers’ awareness, regarding orodental health of their children at age of 1–6 years old. Iran J Nurs.

[20] Esmailikia M, Gholami Parizad E, Abedzadeh Zavareh MS, Sayehmiri K, Ghazanfari Z (2016). Prediction of oral health in children 3–6 years old in Ilam, 2015: application of health belief model. J Ilam Univ Med Sci.

[21] Sim J, Wright C (2000). Research in health care.

[22] Jabarifar SE, Khadem P, Ahmadi S, Hajiahmadi M, Nilchian F (2011). Assessment of psychometric projection of the Persian version of the Child Perception Questionnaire (CPQ8-10) in 8–10 year-old students in Isfahan. J Dent Sch.

[23] Nakamura H, Watanabe N, Matsushima E (2014). Structural equation model of factors related to quality of life for community-dwelling schizophrenic patients in Japan. Int J Ment Health Syst.

[24] Nokhostin MR, Siahkamari A, Akbarzadeh BA (2013). Evaluation of oral and dental health of 6–12 year-old students in Kermanshah city. Iran South Med J.

[25] Dehvari M, Ghaneian MT, Morowatisharifabad MA, Karimi M, Jasemizad T (2018). Knowledge, attitudes and practice of women about adverse effects of cosmetics in Yazd City. Iran Health Scope.

[26] Yavari M, Morowatisharifabad M, Haghi M, Rezaeipandari H, Hatamzadeh N, Azad E (2016). Study of knowledge, attitude, practice and oral health status among high school students in Yazd. Tolooebehdasht.

[27] Naidu R, Nunn J, Donnelly-Swift E (2016). Oral health-related quality of life and early childhood caries among preschool children in Trinidad. BMC Oral Health.

[28] Sahrayi P, Keshavarz Mohammadi N, Ghasemi H (2015). Perceived barriers of mothers in brushing the teeth of their 3–6 years old children, a qualitative study. Iran J Pediatr Dent.

[29] Wong H, McGrath C, King N, Lo E (2011). Oral health-related quality of life in Hong Kong preschool children. Caries Res.

[30] Binkley CJ, Johnson KW. Application of the PRECEDE–PROCEED planning model in designing an oral health strategy. J Theory Pract Dent Public Health 2013;1. PMC4199385 25328904

[31] Rando GM, Jorge PK, Vitor LLR, Carrara CFC, Soares S, Silva TC, Rios D, Machado M, Gavião MB, Oliveira TM (2018). Oral health-related quality of life of children with oral clefts and their families. J Appl Oral Sci Rev FOB.

[32] Saleki M, Jabbarifar SE, Soheilipour S, Hajjizadeh F (2012). Assessing the sensitivity and responsiveness of Early Childhood Oral Health Impact Scale to routine dental treatments on life quality of preschool children in Isfahan in 2011. J Isfahan Dent Sch.

[33] Saho H, Ekuni D (2019). Structural equation modeling to detect predictors of oral health-related quality of life among Japanese university students: a prospective cohort study. Qual Life Res.

