# Supplementary material for: Predictors of oral health-related quality of life in 2–5 year-old children in the South of Iran
Source: Health Qual Life Outcomes. 2020 Dec 11;18:384. doi: 10.1186/s12955-020-01587-7 (PMC7730757; doi:10.1186/s12955-020-01587-7)
Supplement: Supplementary file 1 — Additional file 1. Early Childhood Oral Health Impact Scale (ECOHI). [file 12955_2020_1587_MOESM1_ESM.docx]

ECOHIS questionnaire

| **I do not know** | **Frequently** | **several times** | **Just a few times** | **Very rarely** | **Never** |  | **Number** |
| --- | --- | --- | --- | --- | --- | --- | --- |
|  |  |  |  |  |  | How many times has your child had a toothache, mouth, or jaw pain? | 1 |
|  |  |  |  |  |  | How many times has your child had trouble drinking hot or cold liquids due to oral discomfort or dental treatment? | 2 |
|  |  |  |  |  |  | How many times has your child had difficulty eating due to oral discomfort or dental treatment? | 3 |
|  |  |  |  |  |  | How many times has your child had difficulty pronouncing words due to oral discomfort or dental treatment? | 4 |
|  |  |  |  |  |  | How many times has your child been absent from kindergarten or preschool due to oral or dental problems? | 5 |
|  |  |  |  |  |  | How many times has your child ever had insomnia or lack of sleep due to oral discomfort or dental treatment? | 6 |
|  |  |  |  |  |  | How many times has your child had restlessness or confusion due to oral discomfort or dental treatment? | 7 |
|  |  |  |  |  |  | How many times has your child stopped smiling or laughing because of oral discomfort or dental treatment? | 8 |
|  |  |  |  |  |  | How many times has your child stopped talking due to oral discomfort or dental treatment? | 9 |
|  |  |  |  |  |  | How many times have you or other family members been worried about your child's oral health or dental treatment? | 10 |
|  |  |  |  |  |  | How many times have you or other family members felt guilty about your child's oral problems or dental treatments? | 11 |
|  |  |  |  |  |  | How many times have you or other family members taken time off work or missed work at home due to your child's oral problems or dental treatment? | 12 |
|  |  |  |  |  |  | How many times has your child's oral or dental problems or dental treatment put economic pressure on your family? | 13 |
